# Changes in Microbial Communities Using Pigs as a Model for Postmortem Interval Estimation

**DOI:** 10.3390/microorganisms11112811

**Published:** 2023-11-20

**Authors:** Fan Yang, Xiangyan Zhang, Sheng Hu, Hao Nie, Peng Gui, Zengtao Zhong, Yadong Guo, Xingchun Zhao

**Affiliations:** 1 Institute of Forensic Science, Ministry of Public Security, Beijing 100038, China; yangfan6616@126.com (F.Y.); hsheng2018@163.com (S.H.); oliverepoch@aliyun.com (H.N.); 2 Department of Forensic Science, School of Basic Medical Sciences, Central South University, Changsha 410013, China; zxy196@csu.edu.cn (X.Z.); gdy82@126.com (Y.G.); 3 Department of Microbiology, College of Life Sciences, Nanjing Agricultural University, Nanjing 210095, China; 2019816049@njau.edu.cn (P.G.); ztzhong@njau.edu.cn (Z.Z.)

**Keywords:** postmortem interval estimation, forensic science, microbial community, rupture

## Abstract

Microbial communities can undergo significant successional changes during decay and decomposition, potentially providing valuable insights for determining the postmortem interval (PMI). The microbiota produce various gases that cause cadaver bloating, and rupture releases nutrient-rich bodily fluids into the environment, altering the soil microbiota around the carcasses. In this study, we aimed to investigate the underlying principles governing the succession of microbial communities during the decomposition of pig carcasses and the soil beneath the carcasses. At early decay, the phylum *Firmicutes* and *Bacteroidota* were the most abundant in both the winter and summer pig rectum. However, *Proteobacteria* became the most abundant in the winter pig rectum in late decay. Using genus as a biomarker to estimate the PMI could get the MAE from 1.375 days to 2.478 days based on the RF model. The abundance of bacterial communities showed a decreasing trend with prolonged decomposition time. There were statistically significant differences in microbial diversity in the two periods (pre-rupture and post-rupture) of the four groups (WPG 0–8Dvs. WPG 16–40D, *p* < 0.0001; WPS 0–16Dvs. WPS 24–40D, *p* = 0.003; SPG 0D vs. SPG 8–40D, *p* = 0.0005; and SPS 0D vs. SPS 8–40D, *p* = 0.0208). Most of the biomarkers in the pre-rupture period belong to obligate anaerobes. In contrast, the biomarkers in the post-rupture period belong to aerobic bacteria. Furthermore, the genus *Vagococcus* shows a similar increase trend, whether in winter or summer. Together, these results suggest that microbial succession was predictable and can be developed into a forensic tool for estimating the PMI.

## 1. Introduction

Postmortem interval (PMI) estimation involves the inference and evaluation of the time elapsed between the occurrence of death and the subsequent examination of the deceased body [1]. This pivotal task constitutes a primary and imperative responsibility within the field of forensic practice [2]. The degradation of organic matter by microorganisms represents a fundamental mechanism underlying the process of corpse decomposition [3]. As the decomposer community engages in nutrient recycling, the corpse undergoes a sequence of forensically recognized decomposition stages, encompassing fresh decay, active decay (including bloating and rupture), advanced decay, and skeletonized remains [4]. For the fresh stage, existing methods, such as utilizing body temperature, can provide relatively accurate estimations of the PMI [5]. Nevertheless, in the active decay stage, the body temperature reaches equilibrium with the ambient temperature. Consequently, there remains a pressing necessity to cultivate novel and resilient methodologies that can effectively estimate the PMI beyond the fresh stage.

It is widely recognized that microbiota can be utilized for PMI estimation [6,7,8,9,10,11,12,13,14]. During the fresh stage, cellular macromolecules are released shortly after death, and the microbiota play a crucial role in breaking down these macromolecules into simpler compounds [15]. In the active decay stage, the microbiota engage in anaerobic respiration, generating various gases that contribute to cadaver bloating [15]. Additionally, a significant shift occurs within the microbial community, transitioning from anaerobic to aerobic conditions following the rupture of the abdominal cavity and exposure to the external environment [16]. Zhao et al. suggested that the postmortem oral microbial community data can serve as a forensic resource to estimate the PMI over a long time period, as they found that the abundance of three genera, *Atopostipes*, *Facklamia*, and *Cerasibacillus*, was linearly correlated with PMI in the first 60 days after death [9]. Significant findings have emerged regarding the role of microbial populations in estimating the PMI, which is not exclusive to the oral microbial community [6,9,17,18]. Burcham et al. reported that in murine organs such as the intestines, bone marrow, lungs, and heart, traditionally considered sterile, a notable event occurs during the later stages of decomposition [17]. As the fluids from internal organs begin to emulsify within the decomposing body, colonization by *Clostridium*, a genus of bacteria, can occur. This colonization phenomenon is significant in accurately determining the PMI.

Numerous researchers have conducted comprehensive investigations into microbial succession, focusing predominantly on murine models [8,13,19,20,21,22,23,24,25], specifically rats and mice [2,26]. Metcalf et al. found a “microbial clock” with the capacity to estimate the PMI with a margin of error approximating ±3 days [2], while the experiment was carried out under rigorously controlled circumstances, utilizing experimental mouse models, thus necessitating judicious interpretation when extrapolating these findings to authentic, real-world scenarios [27]. Conversely, Johnson et al., in their exploration involving human subjects, undertook the sampling of the skin microbiome in the context of decomposing human cadavers with an impressive accuracy of approximately ±2 days [28]. Such results represent a substantial advancement when compared to prior methodologies, notably surpassing traditional approaches. Nonetheless, researching PMI inference using human microbiota is constrained by the limited number of body donors, necessitating exploring alternative models. Pigs present a promising option due to their clinical similarities, susceptibility to human enteric pathogens [29], and a remarkable 96% similarity in their gastrointestinal microbiota when compared to humans [30]. Thus, the pig model may offer significant advantages over traditional rodent models for forensic practice [31]. In this study, we selected the pig model to examine postmortem changes in microbial communities, illustrating the potential utility of this approach in forensic science.

Previous decomposition studies have reported a discernible shift in microbial communities, transitioning from a predominance of endogenous gut-associated bacteria before rupture to an increase in non-enteric and aerobic microbes post rupture [16,32]. This observation suggests that accurately determining the temporal proximity to death, both before and following the collapse, can be achieved by monitoring fluctuations in microbial abundance. It further indicates the potential effectiveness of employing a segmentation model to estimate the PMI, comprising the pre-rupture model and the post-rupture model [33]. According to the result of Weiss et al. [31], the “resource selects community”, pre- and post rupture, make the structure of the microbial communities more influencing than the carcass mass [34]. Furthermore, during the rupture stage, releasing nutrient-rich bodily fluids into the environment can lead to an elevation in pH, thereby potentially modifying the endogenous (e.g., intestinal microbiota) and exogenous (e.g., soil microbiota) microbial communities [2,15,35]. Although this phenomenon has been found in previous studies, a detailed comparative analysis has not been conducted, and there are few reports on whether seasonal factors will affect microorganisms pre- and post rupture.

Hence, this study offers a high-throughput sequencing-based characterization of bacterial communities inhabiting the rectum of pigs and the surrounding corpse-associated soils in winter and summer. The aim is to compare bacterial temporal dynamics between the pre- and post-rupture stages under different seasons, finding the significant biomarkers that could be used regardless of the season, thereby enhancing our understanding of bacterial communities and providing new insight into the translation of animal-derived data to human conditions, thereby offering valuable insights for future research.

## 2. Materials and Methods

### 2.1. Experimental Design and Sample Collection

The procedures involving animal care in this study were approved by the Animal Care and Use Committee of the Nanjing Agricultural University (Nanjing, China) (permit number: SYXK(Su)2017-0007). This study obtained six domestic pigs from the Qinglongshan animal farm, Jiangning, Nanjing, China, divided into summer (n = 3) and winter groups (n = 3). After inducing anesthesia with ether, the head of the pigs received a precise strike using a blunt instrument to ensure a humane and swift death. The corpse was placed in a pre-dug pit and covered with soil for a simulated burial environment in a forest of Xuanwu, Nanjing, Jiangsu, China (32°04 N, 118°50 E).

**Figure 1:**
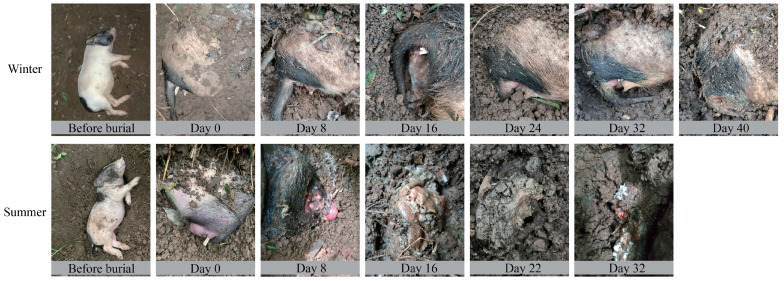
Morphological changes of pigs buried in different seasons.

Rectal and grave soil samples were collected. (1) Rectal sample: The sterile swab was inserted into the rectum, rotated gently, and removed. Three samples were collected by repeating the step. (2) Grave soil sample: While minimizing disturbance to the overall structure of the grave, a soil sampler was used to collect soil samples from the grave. The collected soil was divided into three portions. After collecting the sample, the grave was covered using the excavated soil. The experiment lasted 40 days for the winter group, and samples were collected on day 0, 8, 16, 24, 32, and 40. The experiment lasted 32 days for the summer group, and samples were collected on day 0, 8, 16, 22, and 32 (due to weather conditions, the sampling on day 24 of summer was not carried out, and the sample on day 22 was selected as an alternative) (Figure 1). The day 0 samples of soil are the initial soil samples. All samples were frozen at −80 °C until further utilization. A total of 72 samples were collected.

### 2.2. DNA Extraction, PCR Amplification, and Sequencing

The genomic DNA was extracted using the E.Z.N.A. Soil DNA Kit (Omega Bio-tek, Inc., Norcross, GA, USA) following the manual. The concentration and quality of the genomic DNA were checked using a NanoDrop 2000 spectrophotometer (Thermo Scientific Inc., Waltham, MA, USA). DNA samples were stored at −20 °C for subsequent experiments.

The V3-4 hypervariable region of the bacterial 16S rRNA gene was amplified with the universal primers 341F (5′-CCTAYGGGRBGCASCAG-3′) and 806R (5′-GGACTACNNGGGTATCTAAT-3′), to which barcode sequences were added for the Illumina MiSeq sequencing. The PCR reaction mixture was prepared using 2× Taq PCR MasterMix (Vazyme Biotech Co., Ltd., Nanjing, China). The PCR products were purified and qualified using an Agencourt AMPure XP Kit (Beckman Coulter, Inc., Pasadena, CA, USA), Nanodrop 2000 (ThermoFisher Scientific, Inc., Waltham, MA, USA), Agilent 2100 Bioanalyzer (Agilent Technologies, Inc., Santa Clara, CA, USA), and ABI StepOnePlus Real Time PCR System (Applied Biosystems, Inc., Waltham, MA, USA). Then, the PCR products were paired-end sequenced with the Illumina Miseq PE300 platform (Illumina Company, San Diego, CA, USA).

### 2.3. Data Analysis and Processing

High-throughput sequencing reads were merged and quality-filtered using a combination of Pear (v0.9.6), Vsearch (v2.7.1), and uchime. Qualified sequences were clustered into operational taxonomic units (OTUs) at a similarity threshold of 97%. QIIME (v1.8.0) was used to generate rarefaction curves and calculate the richness and diversity indices based on the OTU information. An alpha diversity (Chao1, Shannon, and Simpson indexes) analysis was performed using QIIME (v1.8.0). A principal component analysis (PCA) and a non-metric multidimensional scaling analysis (NMDS) were performed to visualize distance matrices and evaluate differences among different groups. The random forest (RF) model was used to predict the PMI based on bacterial taxa abundances. Biomarkers were selected through a 10-fold cross-validation to minimize errors. Additionally, a RF regression model was established to improve the PMI prediction accuracy, measured using the mean absolute error (MAE) and coefficient of determination (R^2^). A linear discriminant analysis effect size (LEfSe) was used to find the biomarkers between different groups. All analyses were carried out using R packages (v3.6.0)and SIMCA 14.1.

## 3. Results

### 3.1. Bacterial Community Structure Overview

**Figure 2:**
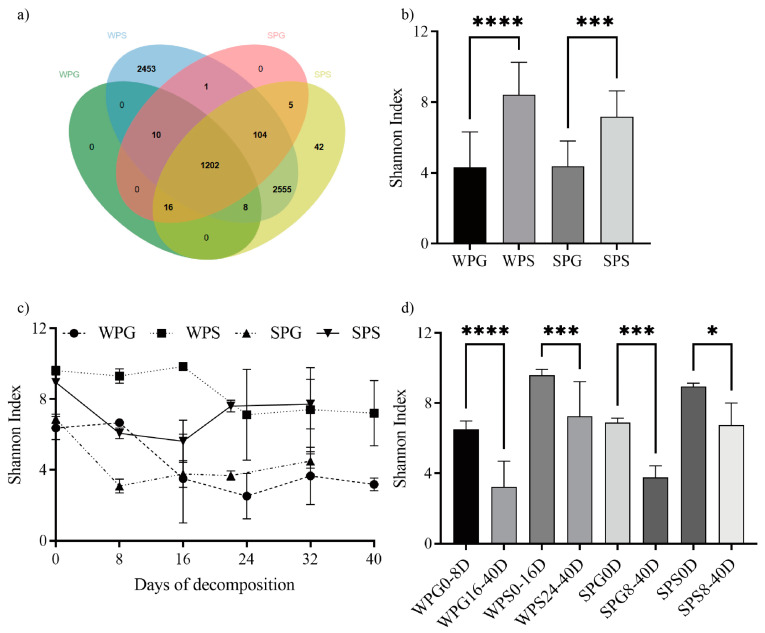
An overview of the differences between groups. (**a**) Venn of OTUs in different groups; green for WPG, blue for WPS, pink for SPG, and yellow for SPS; WPG: winter pig rectal sample; WPS: winter pig soil sample; SPG: summer pig rectal sample; SPS: summer pig soil sample. (**b**) The Shannon index of different groups. (**c**) The Shannon index tendency over time as the corpse decomposed. (**d**) The difference between groups when dividing the sample into two stages. *: *p* < 0.05, ***: *p* < 0.001, ****: *p* < 0.0001.

By performing high-throughput sequencing, 2,992,761 raw reads and 2,896,934 clean reads were obtained. The Shannon–Wiener and species accumulation curves became smooth and stable, indicating that our sequencing depth was adequate for all samples (Figure S1). The ratio of clean reads to raw reads was 96.80%. The length of most high-quality sequences (98.56%) ranged from 400 to 440. A total of 6396 OTUs were clustered based on 97% similarity, containing 1346 OTUs of the rectum sample and 6396 OTUs of the soil sample (Table 1). A Venn diagram was plotted to compare the similarities and variances among the communities obtained in the different groups, which showed that most OTUs in pig rectums can be detected in soil (Figure 2a). There was little difference in seasonal OTUs both in the rectal and soil samples.

### 3.2. Alpha Diversity and Beta Diversity

The alpha diversity indexes (chao1, goods coverage, observed species, PD whole tree, Shannon, and Simpson) are shown in Table S1. The bacterial diversity of the soil sample was higher than the rectal sample, whether in summer (*p* = 0.0002, one-way ANOVA) or winter (*p* < 0.0001, one-way ANOVA), using the Shannon index, as shown in Figure 2b. At the same time, there was no significant difference in bacterial diversity of the rectal sample (*p* > 0.9999, one-way ANOVA) between winter and summer, and the same was true for the soil sample (*p* = 0.2436, one-way ANOVA). Figure 2c shows that the Shannon index decreased over time as the corpse decomposed. The apparent decrease was found between day 8 and day 16 of the winter pig rectal sample (WPG), between day 16 and day 24 of the winter pig soil sample (WPS), and between day 0 and day 8 of both the summer pig rectal sample (SPG) and summer pig soil sample (SPS), as shown in Figure 2c. Figure 2d was drawn to clarify whether there was a statistical difference between the periods before and after the decline in bacterial diversity. There were statistically significant differences in bacterial diversity in the two periods of the four groups (WPG 0–8D vs. WPG 16–40D: *p* < 0.0001, WPS 0–16D vs. WPS 24–40D: *p* = 0.003, SPG 0D vs. SPG 8–40D: *p* = 0.0005, and SPS 0D vs. SPS 8–40D: *p* = 0.0208).

**Figure 3:**
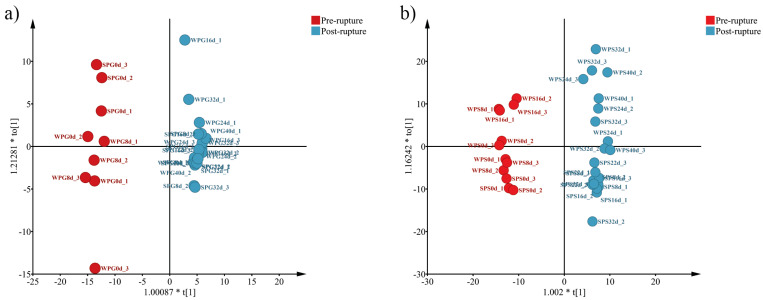
OPLS-DA using the genus abundance. (**a**) The score scatter plot of rectum samples; (**b**) The score scatter plot of soil samples. WPG: winter pig rectal sample; WPS: winter pig soil sample; SPG: summer pig rectal sample; SPS: summer pig soil sample.

According to the NMDS results obtained with Bray–Curtis distances (Figure S2), the bacterial communities of all groups (all stress < 0.2) formed two discrete clusters corresponding to the early and late stages consistent with the alpha diversity results. The same result could also be found in the analysis of OPLS-DA, which only uses the genus abundance (Figure 3). As Figure 3a shows, all rectum samples could be divided into two clusters, pre-rupture (WPG0–8D and SPG0D) and post-rupture (WPG16–40D and SPG8–40D), regardless of the seasons, and the same was true for the soil samples (Figure 3b).

### 3.3. Taxonomic Analysis

**Figure 4:**
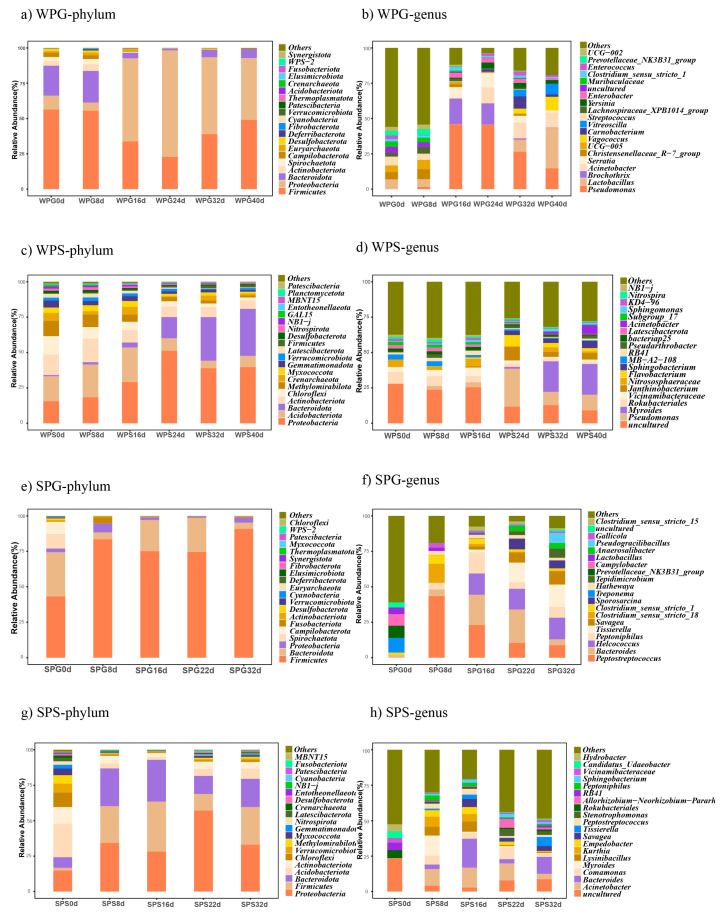
The relative abundance of bacteria in different groups changes with decomposition. (**a**) The phylum level of WPG; (**b**) the genus level of WPG; (**c**) the phylum level of WPS; (**d**) the genus level of WPS; (**e**) the phylum level of SPG; (**f**) the genus level of SPG; (**g**) the phylum level of SPS; and (**h**) the genus level of SPS. WPG: winter pig rectal sample; WPS: winter pig soil sample; SPG: summer pig rectal sample; SPS: summer pig soil sample.

The microorganisms could be classified into 53 phyla, 133 classes, 319 orders, 502 families, 1070 genera, and 1093 species. Both in winter and summer, the soil bacterial abundance was higher than the rectum bacterial abundance at any taxonomic level, especially at genera and species. The top five phyla of WPG are *Firmicutes* (42.80%), *Proteobacteria* (41.32%), *Bacteroidota* (10.00%), *Campilobacterota* (1.36%), and *Spirochaetota* (1.25%) (Figure 4a), and the same is true for SPG with *Firmicutes* (73.51%), *Bacteroidota* (17.25%), *Proteobacteria* (3.12%), *Spirochaetota* (2.08%), and *Campilobacterota* (1.69%) (Figure 4c). The top five phyla of WPS are *Proteobacteria* (32.20%), *Acidobacteriota* (14.55%), *Bacteroidota* (14.19%), *Actinobacteriota* (10.03%), and *Chloroflexi* (5.88%) (Figure 4e), while the top five in SPS are *Proteobacteria* (33.54%), *Firmicutes* (20.32%), *Bacteroidota* (19.23%), *Acidobacteriota* (8.18%), and *Actinobacteriota* (5.99%) (Figure 4g). The top five genera of WPG are *Pseudomonas* (22.41%), *Lactobacillus* (8.26%), *Brochothrix* (5.75%), *Acinetobacter* (5.66%), and *Serratia* (3.97%) (Figure 4b). The top five genera of SPG are *Peptostreptococcus* (17.12%), *Bacteroides* (11.08%), *Helcococcus* (8.95%), *Peptoniphilus* (6.37%), and *Tissierella* (6.35%) (Figure 4d). The top five genera of WPS, excluding uncultured ones, are *Pseudomonas* (8.90%), *Myroides* (7.44%), *Rokubacteriales* (4.19%), *Vicinamibacteraceae* (3.19%), and *Janthinobacterium* (3.08%) (Figure 4f). The top five genera of SPS, excluding uncultured ones, are *Acinetobacter* (8.31%), *Bacteroides* (7.78%), *Comamonas* (4.18%), *Myroides* (3.29%), and *Lysinibacillus* (2.90%) (Figure 4h).

### 3.4. Utilizing Bacterial Communities to Predict the PMI Based on the Model of RF

**Figure 5:**
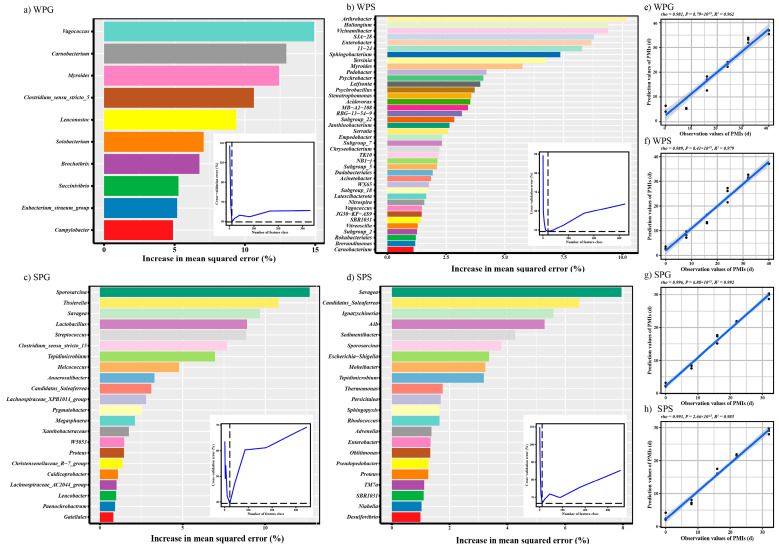
The biomarkers and RF model based on genus. (**a**–**d**) The top biomarker bacterial genera were identified by applying a random forest model of their relative abundances in each group against PMIs. Biomarker taxa are ranked in descending order of importance to the accuracy of the model. The inset represents a 10-fold cross-validation error as a function of the number of input genera used to regress against the PMI. (**a**) WPG; (**b**) WPS; (**c**) SPG; (**d**) SPS; (**e**–**h**) RF model based on genus. (**e**) WPG; (**f**) WPS; (**g**) SPG; (**h**) SPS.

To process the large datasets obtained using high-throughput sequencing, we regressed the relative abundance of bacterial communities at the genus level against the PMl using the RF machine learning algorithm for each group (Figure 5). A 10-fold cross-validation was performed to reveal the importance of bacterial genera as biomarker taxa during cadaver decomposition. The top 10 genera were chosen to estimate the PMI for WPG with MAE = 2.478 days (*rho* = 0.981, *R*^2^ = 0.962). The top 40 genera were chosen to estimate the PMI for WPS with MAE = 2.001 days (*rho* = 0.989, *R*^2^ = 0.979). The genera *Vagococcus*, *Myroides*, and *Carnobacterium* are significant in estimating the PMI in winter grave soil and carcasses. The top 22 genera were chosen to estimate the PMI for SPG with MAE = 1.375 days (*rho* = 0.996, *R*^2^ = 0.992). The top 22 genera were chosen to estimate the PMI for SPS with MAE = 1.567 days (*rho* = 0.993, *R*^2^ = 0.985). The genera *Proteus*, *Candidatus_Soleaferrea*, *Tepidimicrobium*, *Savagea*, and *Sporosarcina* are significant in estimating the PMI in summer grave soil and carcasses. However, according to the result of RF, there were no identical biomarkers for estimating the PMI between winter and summer pig carcasses. *SBR1031* (*Chloroflexi* phylum) and *Enterobacter* are significant in estimating the PMI both in summer and winter grave soil.

### 3.5. Bacterial Difference between Pre-Rupture and Post-Rupture Groups

**Figure 6:**
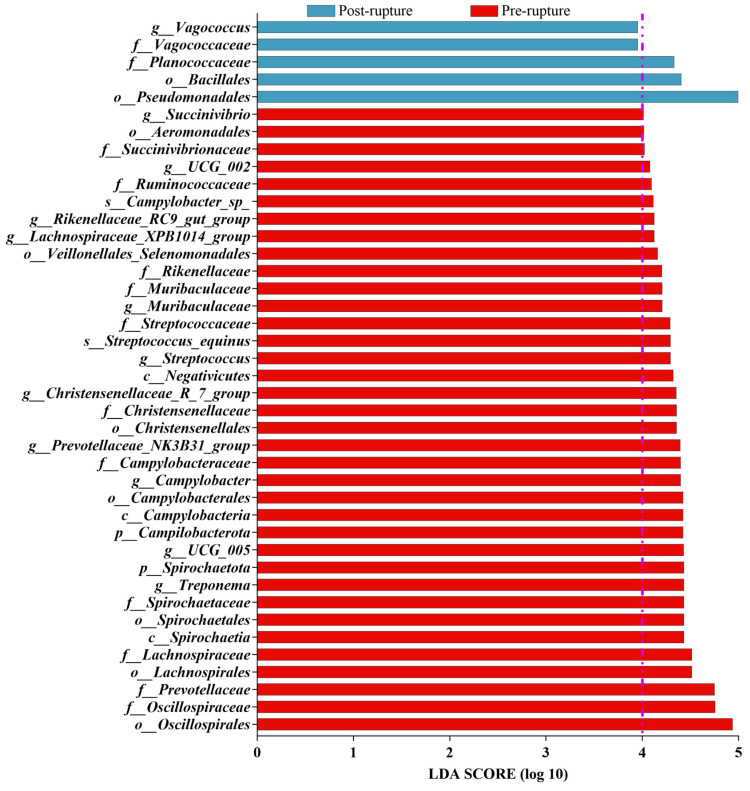
The biomarkers obtained using LEfSe analysis with an LDA > 4 in the rectum, including the genus *Vagococcus* and family *Vagococcaceae* with an LDA score of 3.96. The purple dotted line means LDA = 4.

**Figure 7:**
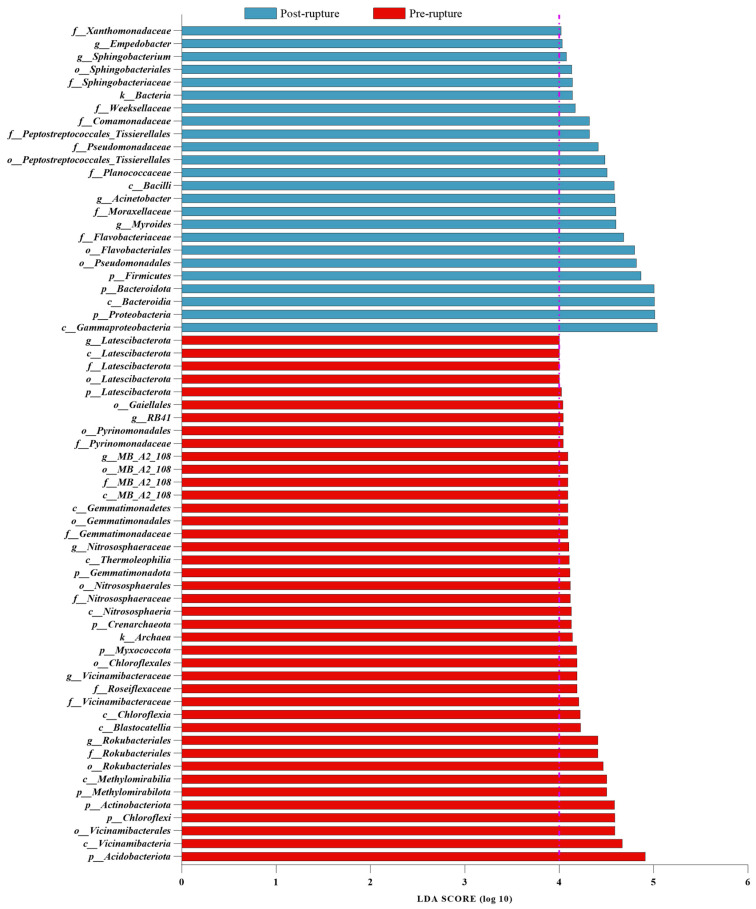
The biomarkers obtained using LEfSe analysis with an LDA > 4 in the soil. The purple dotted line means LDA = 4.

According to the alpha diversity and beta diversity results, the samples could be divided into two groups: pre- and post-rupture. LEfSe (linear discriminant analysis effect size) was employed to conduct biomarker analysis between these two groups, disregarding the seasonal factor (We conducted distinct analyses for each of the three datasets: summer dataset, winter dataset, and combined summer and winter datasets. The final result entailed identifying their intersection.) (Figures S3–S6). For the pig rectal sample: The genus *Treponema* exhibited relevance to the pre-rupture stage with an LDA score of 4.43, whereas *Vagococcus* showed an increase in the post-rupture phase with an LDA score of 3.96. For the pig soil sample: The genus *Rokubacteriales* displayed relevance to the pre-rupture stage with an LDA score of 4.41, whereas *Myroides* showed an increase in the post-rupture phase with an LDA score of 4.60. All bacteria with an LDA score > 4 are shown in Figure 6 and Figure 7, including the genus *Vagococcus* and family *Vagococcaceae* with an LDA score of 3.96.

## 4. Discussion

This study examined the variations in the bacterial communities within the cadavers of domestic pigs during the summer and winter decay process. It reveals noticeable disparities in alpha diversity between the burial soil and the pig rectal region, demonstrating a decreasing trend with prolonged decomposition time. Furthermore, bacterial community structures at different stages of decomposition within the same sample exhibit distinct differences, thereby affirming the significance of time and sampling location as pivotal factors influencing bacterial community dynamics during the decay process of domestic pig cadavers.

At early decay, the phyla *Firmicutes* and *Bacteroidota* were the most abundant in both the winter and summer pig rectum. However, *Proteobacteria* became the most abundant phylum in the winter pig rectum in late decay. The increase in *Proteobacteria* could be found in other research [36]. As Procopio et al. claimed, *Proteobacteria* were the most abundant phylum within most experimental samples without insects after prolonged decomposition stages [36]. The reason why *Firmicutes* were still the most abundant phylum in the summer pig rectum may be due to temperature and insects. According to the same experiments conducted in summer, *Firmicutes* increased with prolonged PMIs, as found by Pechal et al. [16]. Furthermore, it is worth noting that our experimental design may not have effectively prevented insects, including flies, from gaining access to our carcasses. This potential lack of exclusion could account for the observed differences between the two seasons, particularly given the reduced insect activity during the winter months. Nonetheless, it is important to highlight that despite these seasonal variations, our analysis of the Shannon index indicates no significant difference in bacterial diversity in either the soil or rectal samples across the seasons.

A discernible shift in bacterial communities was observed between pre- and post-rupture according to NMDS (Figure S2) and alpha diversity results (Figure 2b,d). Rupture is a crucial stage during decomposition, in which bloating due to putrefaction breaks open the abdominal cavity. It is expected to result in bacterial community shifts because the cavity becomes aerobic. Using OPLS-DA to analyze the abundance of the genera (Figure 3a) also found that all groups could be divided into two clusters, pre-rupture (WPG0–8D and SPG0D) and post-rupture (WPG16–40D and SPG8–32D), which indicated that the effect of rupture on bacterial abundance was more significant than the season factor. Moreover, nutrient-rich bodily fluids released into the environment at the rupture stage could alter soil bacterial communities. This could also be confirmed by our result (Figure 3b), which shows two clusters with significant differences: pre-rupture (WPS0–16D and SPS0D) and post-rupture (WPS24–40D and SPS8–32D). The observed delay in the shift of soil bacterial communities compared to the rectal bacterial communities may be attributed to the time required for cadaver decomposition consequences to permeate the surrounding environment. Consequently, the change in the composition of soil bacterial communities may lag behind the changes observed in the rectal samples.

To avoid the temperature and/or insect potential effect, we use the intersection set of two seasons of bacteria to discuss the difference between pre- and post-rupture, aiming to find the significant biomarkers that could be suitable for different seasons. Table 2 shows that the significant biomarkers in the rectum and soil are dissimilar at the pre-rupture stage, in which the 94 biomarkers in the soil did not overlap with the 34 biomarkers in the rectum, according to the result of LEfSe. One possible reason is that the microbes in the gut and the environmental soil are different before and after rupture, resulting in different characteristic microbes. For the post-rupture stage, the number of significant biomarkers is far lower than for the pre-rupture stage, and only three phyla, *Proteobacteria*, *Bacteroidota*, and *Firmicutes*, show statistical significance in the soil, while no significant phylum or class was found in the rectum. Unlike pre-rupture, the significant biomarkers tend to be the same for soil and rectum at the post-rupture stage. For example, the abundance of the order *Pseudomonadales* and the family *Planococcaceae* increase in these samples at the post-rupture stage. The result is the same as in the late postmortem changes, in which the soft tissue breaks down in the post-putrefaction period, penetrating the soil, resulting in a soil environment similar to that of the cadaver, and the bacterial communities from the cadaver mix with the soil.

We also found that most of the biomarkers in the pre-rupture stage belong to obligate anaerobes. In contrast, the biomarkers in the post-rupture stage belong to aerobic bacteria (facultative anaerobes could be found as biomarkers in both these stages). The possible reason for the shift may be oxygen entry, enabled due to body rupture. A primary literature search showed that facultative anaerobes (e.g., *Lactobacillus*) have been shown to increase at the bloating stage but decrease greatly after the rupture stage [37]. Our results also provided support for these findings (Figure 4). As mentioned earlier, the abundance of the order *Pseudomonadales* and the family *Planococcaceae* increased with decay time. Both of them belong to aerobic bacteria. This result is similar to those reported before [11,38]. As Tozzo et al. reported, the predominant bacterial families in advanced decomposition stages included *Pseudomonadaceae* and *Planococcaceae* [11]. Similarly, Karoline et al. found that the order *Pseudomonadales* was present at all time points on corpses but was especially abundant on days 3–14 [38].

The *Vagococcus* genus, which includes aerobic bacteria, is the only significant biomarker at the post-rupture stage in the winter and summer group, whose abundance also increases with decay time. The *Vagococcus* genus could be isolated from the soil under a decaying pig carcass, suggesting that *Vagococcus* sp. might facilitate further cadaver decomposition [39]. The same was reported by Johnson et al., as the genus *Vagococcus* was identified as an important feature in estimating the PMI using machine learning methods [28], given that its abundance increased significantly in the late postmortem stage.

The present investigation comprehensively analyzed temporal shifts in microbial communities associated with pig cadavers, indicating significant differentiations in microbiota composition between the pre- and post-rupture groups. Given the pronounced disparities in decomposition stages, future studies should methodically incorporate this pivotal rupture point into their methodologies. To establish a precise and robust PMI prediction model grounded in bacterial ecological succession, adopting a bimodal modelling approach is essential, encompassing both classification and regression models. Moreover, focusing on biological fluids such as saliva and skin should also be considered in future PMI studies, like the studies of geographic location identification and bodily fluid discrimination [40,41].

## 5. Conclusions

In this study, we comprehensively examined the gut bacterial community in pig carcasses, revealing a consistent and predictable succession of bacterial abundance. The pre-and post-rupture stages are based on the relative abundance of bacterial communities in both winter and summer. Additionally, we identified bacterial biomarkers under different seasonal conditions to distinguish between the two stages. While pigs and humans share similarities in their bacterial communities, it is imperative to underscore the need for further validation by collecting and analyzing human samples to corroborate our findings in future investigations.

## Figures and Tables

**Table 1:** Sequence results after quality control.

Time	Sample	Raw Sequence Number	Clean Sequence Number	OTUs
Summer	Rectum	670007	652672	1338
	Grave soil	663546	643916	3932
Winter	Rectum	800908	772761	1236
	Grave soil	858300	827585	6333

**Table 2:** Top five significant biomarkers in the rectum and soil according to LEfSe.

Stage	Sample	Phylum	Class	Order	Family	Genus
Pre-rupture	rectum	*Spirochaetota*	*Spirochaetia*	*Oscillospirales*	*Oscillospiraceae*	*Treponema*
		*Campilobacterota*	*Campylobacteria*	*Lachnospirales*	*Prevotellaceae*	*UCG_005*
		*Desulfobacterota*	*Negativicutes*	*Spirochaetales*	*Lachnospiraceae*	*Campylobacter*
		*Deferribacterota*	*Desulfovibrionia*	*Campylobacterales*	*Spirochaetaceae*	*Prevotellaceae_NK3B31_group*
			*Deferribacteres*	*Christensenellales*	*Campylobacteraceae*	*Christensenellaceae_R_7_group*
	soil	*Acidobacteriota*	*KD4_96*	*Frankiales*	*Nitrospiraceae*	*Subgroup_25*
		*Chloroflexi*	*MB_A2_108*	*Latescibacterota*	*Rokubacteriales*	*MND1*
		*Actinobacteriota*	*TK10*	*Haliangiales*	*MB_A2_108*	*Subgroup_5*
		*Methylomirabilota*	*NB1_j*	*Subgroup_25*	*Vicinamibacteraceae*	*KD4_96*
		*Myxococcota*	*Vicinamibacteria*	*Nitrospirales*	*Haliangiaceae*	*11_24*
Post-rupture	rectum	*-*	*-*	*Pseudomonadales*	*Vagococcaceae*	*Vagococcus*
				*Burkholderiales*	*Planococcaceae*	
				*Bacillales*		
	soil	*Proteobacteria*	*Gammaproteobacteria*	*Pseudomonadales*	*Flavobacteriaceae*	*Myroides*
		*Bacteroidota*	*Bacteroidia*	*Flavobacteriales*	*Moraxellaceae*	*Acinetobacter*
		*Firmicutes*	*Bacilli*	*Peptostreptococcales_Tissierellales*	*Planococcaceae*	*Sphingobacterium*
				*Sphingobacteriales*	*Pseudomonadaceae*	*Empedobacter*
				*Enterobacterales*	*Peptostreptococcales_Tissierellales*	*Stenotrophomonas*

## Data Availability

The raw sequence data can be found in NCBI (PRJNA1020278).

## Supplementary Materials

The following supporting information can be downloaded at: https://www.mdpi.com/article/10.3390/microorganisms11112811/s1, Figure S1: The Shannon–Wiener and species accumulation curves; Figure S2: The NMDS results obtained with Bray–Curtis distances; Figure S3: The LEfSe result of WPG; Figure S4: The LEfSe result of WPS; Figure S5: The LEfSe result of SPG; Figure S6: The LEfSe result of SPS; Table S1: Alpha diversity index statistical table.
