# Supplementary material for: Changes in Microbial Communities Using Pigs as a Model for Postmortem Interval Estimation
Source: Microorganisms. 2023 Nov 20;11(11):2811. doi: 10.3390/microorganisms11112811 (PMC10672931; doi:10.3390/microorganisms11112811)
Supplement: Supplementary file 1 [file microorganisms-11-02811-s001.zip › Figure S1.pdf]

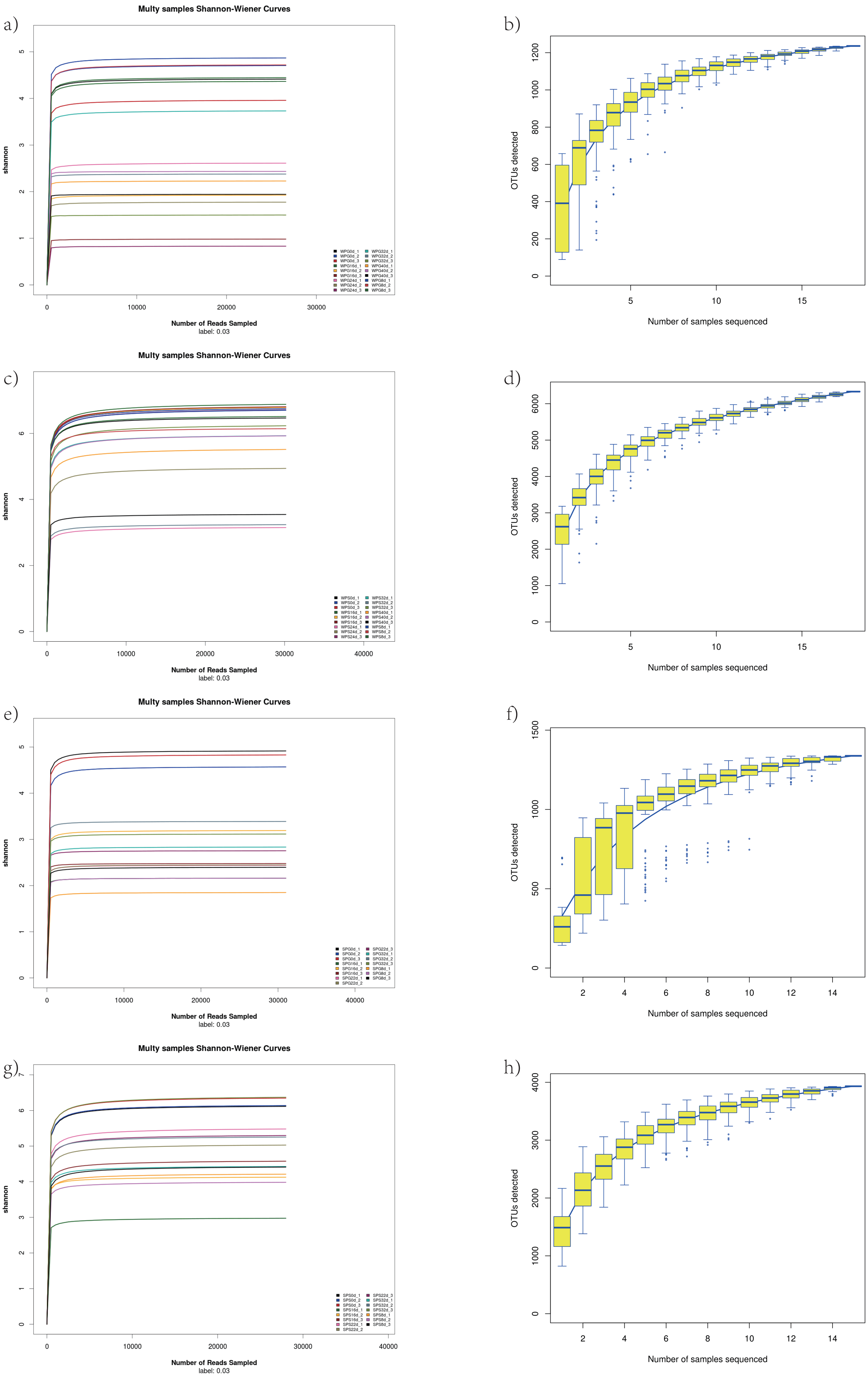
a)
Multy samples Shannon-Wiener Curves
shannon
Number of Reads Sampled
label: 0.03
WPG0d_1
WPG0d_2
WPG0d_3
WPG16d_1
WPG16d_2
WPG16d_3
WPG24d_1
WPG24d_2
WPG24d_3
WPG32d_1
WPG32d_2
WPG32d_3
WPG40d_1
WPG40d_2
WPG40d_3
WPG8d_1
WPG8d_2
WPG8d_3
b)
OTUs detected
Number of samples sequenced
c)
Multy samples Shannon-Wiener Curves
shannon
Number of Reads Sampled
label: 0.03
WPS0d_1
WPS0d_2
WPS0d_3
WPS16d_1
WPS16d_2
WPS16d_3
WPS24d_1
WPS24d_2
WPS24d_3
WPS32d_1
WPS32d_2
WPS32d_3
WPS40d_1
WPS40d_2
WPS40d_3
WPS8d_1
WPS8d_2
WPS8d_3
d)
OTUs detected
Number of samples sequenced
e)
Multy samples Shannon-Wiener Curves
shannon
Number of Reads Sampled
label: 0.03
SPG0d_1
SPG0d_2
SPG0d_3
SPG16d_1
SPG16d_2
SPG16d_3
SPG22d_1
SPG22d_2
SPG22d_3
SPG32d_1
SPG32d_2
SPG32d_3
SPG8d_1
SPG8d_2
SPG8d_3
f)
OTUs detected
Number of samples sequenced
g)
Multy samples Shannon-Wiener Curves
shannon
Number of Reads Sampled
label: 0.03
SPS0d_1
SPS0d_2
SPS0d_3
SPS16d_1
SPS16d_2
SPS16d_3
SPS22d_1
SPS22d_2
SPS22d_3
SPS32d_1
SPS32d_2
SPS32d_3
SPS8d_1
SPS8d_2
SPS8d_3
h)
OTUs detected
Number of samples sequenced
