# Supplementary figures and images for: Changes in Microbial Communities Using Pigs as a Model for Postmortem Interval Estimation
Source: Microorganisms. 2023 Nov 20;11(11):2811. doi: 10.3390/microorganisms11112811 (PMC10672931; doi:10.3390/microorganisms11112811)

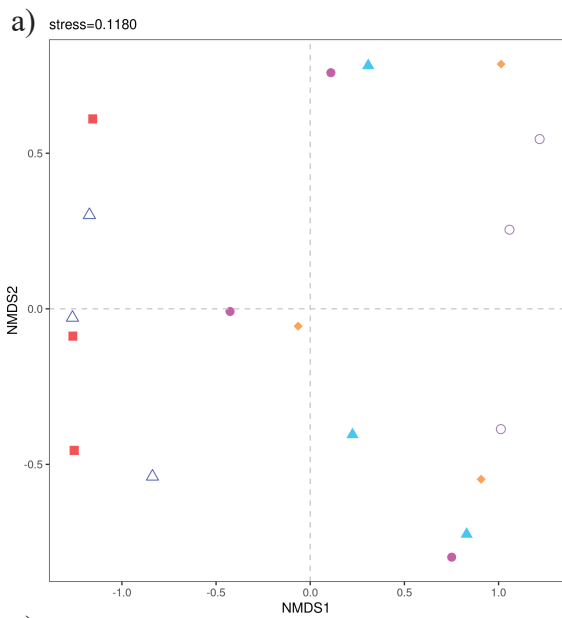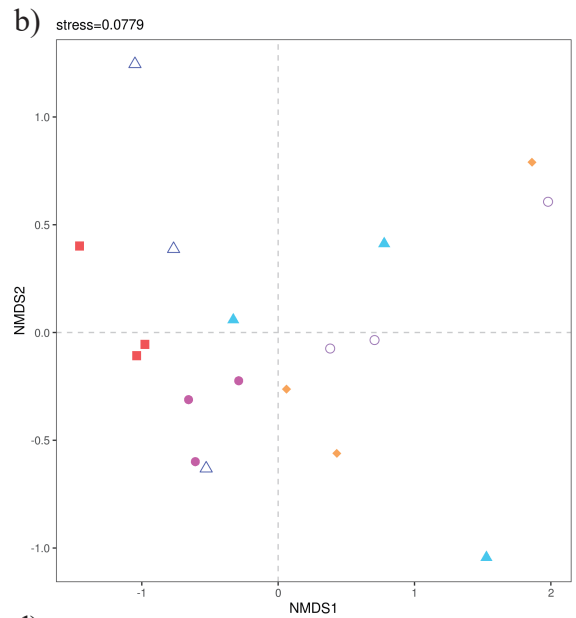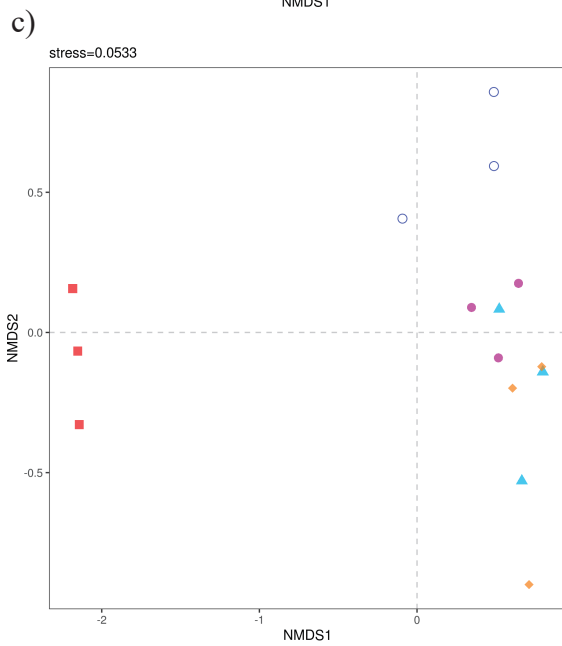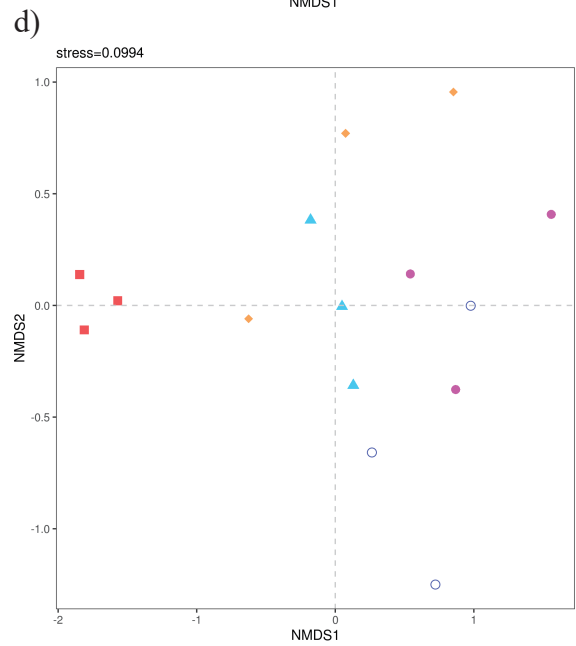

Supplement: Supplementary file 1 [file microorganisms-11-02811-s001.zip › Figure S2.pdf]
